# Fungal succession during mammalian cadaver decomposition and potential forensic implications

**DOI:** 10.1038/s41598-019-49361-0

**Published:** 2019-09-09

**Authors:** Xiaoliang Fu, Juanjuan Guo, Dmitrijs Finkelbergs, Jing He, Lagabaiyila Zha, Yadong Guo, Jifeng Cai

**Affiliations:** Department of Forensic Science, School of Basic Medical Sciences, Central South University, Changsha, Hunan 410013 China

**Keywords:** Next-generation sequencing, Fungal ecology

## Abstract

The necrobiome is the postmortem community that includes bacteria, fungi, arthropods, and other cadaver-associated organisms. It has been suggested as biological evidence for forensic investigation. Fungi form distinctive mildew spots in colonizing decomposing bodies, converting them into moldy cadavers. However, the postmortem fungal community consists of more than these visible species. Characterizing the succession pattern of the fungal community during decomposition is valuable not only for understanding the ecosystem composition of the cadaver decomposition islands but also for contributing to forensic investigations. In the present study, the fungal composition of pig cadavers and succession patterns during decomposition were investigated with high-throughput sequencing. The succession patterns were easier to discern in outdoor cadavers, compared with those that were placed indoors. The metabarcoding approach revealed trends linking particular fungal taxa with specific postmortem intervals (PMIs). Dominant species increased notably in cadavers and soil. Furthermore, the succession of the soil community was driven by the cadaver decomposition. Significant mycoflora differences were observed between environmental and cadaveric soil. The results obtained suggested that postputrefaction mycoflora have considerable potential for PMI estimation, particularly in cases that involve heavily decomposed bodies. In addition, the diversity of fungal communities revealed by the metabarcoding approach allowed us to discriminate the sites of cadaver decomposition, implying that postputrefaction mycoflora may be helpful in identifying the environment in which a cadaver has been placed, or the original location from which a cadaver has been moved. Our results provide an important step towards developing fungal evidence for use in forensic science and add to the growing body of work on postmortem microbial communities.

## Introduction

Cadaver decomposition is a universal phenomenon which plays an integral role in ecosystem energy transformation and nutrient cycling^1^. The complex process of decomposition of human or other mammalian cadavers is intimately affected by biotic (bacteria, fungi, arthropods, nematodes, etc.) and abiotic (weather, climate, temperature, humidity, etc.) factors^2^. As a pool of detritus, cadavers are a much more concentrated source of nutrients (low carbon to nitrogen ratio, high water content) than plant litter^3^. Without the action of microbes, chemical decomposition would proceed at an extremely slow pace, leading to the formation of reservoirs of biochemical waste^4^. These cadaver-associated microbes are part of the necrobiome^5^, which is derived from the microbial communities that inhabited the live host^6^ and the environment where the cadaver falls^7^. Previous studies have shown that postmortem bacteria are derived primarily from the soil and significantly impact the rate at which cadavers decompose, while the key decomposers are ubiquitous in low abundance^1,8,9^. Identifying the microflora involved in cadaver decomposition can provide a detailed understanding into how the biological community changes over time^10^. Although many studies have described spatial and temporal variation in bacterial communities during decomposition^11–13^, our understanding of the succession pattern of postputrefaction mycoflora remains limited.

Fungi can colonize decomposed bodies, forming distinctive mildew spots, ultimately converting bodies into moldy cadavers at the dry stage of decomposition^14,15^. Heavily decomposed cadavers, in particular those that are highly mummified, often present visible fungal growth^16^. Artificial cultivation has allowed for the morphological identification of the fungi that tend to colonize cadavers. *Aspergillus*, *Penicillium*, *Candida* and *Mucor* have been identified as the dominant genera in the bloat, putrefaction, and skeletonization stages^17^. Using molecular approaches, numerous species in the genera listed above were identified among samples collected during 23 autopsies, performed on body surfaces with visible fungal growth^16^. Furthermore, saprophytic genera such as *Monilia*, *Penicillium*, *Alternaria*, *Aspergillus*, *Rhizopus*, *Chrysosporium*, and *Cladophialophora* have been isolated from mummified cadavers in archeological studies^18,19^. However, the roles of yeasts and other unicellular fungi in postmortem communities remain to be characterized^9,20,21^. Information pertaining to the composition of the fungal community during decomposition is valuable for various aspects of forensic investigation, such as estimations of postmortem interval (PMI), postburial interval (PBI), location of clandestine graves, and other efforts to characterize the environment in which the cadaver is located^14,22–24^. However, technical biases continue to limit visualization of the fungal community structure. There is an ongoing need to develop new methods to fully characterize the succession pattern of postmortem fungi.

In the present study, juvenile pigs were used as human analogues to establish decomposition models under both indoor and outdoor settings. The outdoor cadavers were placed directly in fields and exposed to the surrounding soil and insects. Indoor cadavers were placed in cabins and segregated from outside factors. The fungal communities from the cadavers and soil were harvested at different time points for high-throughput sequencing (HTS) analysis. We expected that the cadaveric communities would show notable and repeatable succession patterns throughout the decomposition process, and that the dominant species would change over time during the PMI. In addition, we compared the fungal communities that accumulated on cadaver surfaces to reveal dissimilarities between indoor and outdoor cadavers. Similar to previous studies that have soil fungi as indicators to estimate PBI^9,23^, we also explored the succession pattern of soil fungi underneath cadavers. Furthermore, the effects of decomposition on soil fungi have been assessed by comparing the communities of environmental and cadaveric soil. The results obtained allowed us to delineate the patterns of fungal succession during indoor and outdoor decomposition. Based on our findings, we propose the use of cadaver-associated fungi as microbial evidence in several aspects of forensic investigation.

## Results

### Fungal succession in indoor cadavers

**Figure 1:**
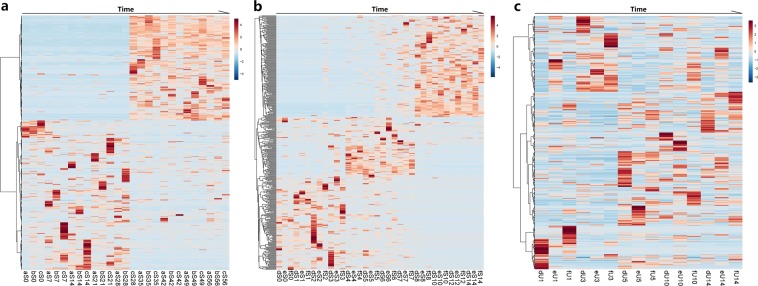
Heat maps for indoor cadaveric communities (**a**), outdoor cadaveric communities (**b**), and soil communities underneath the cadavers. (**c**) The samples on the X-axis are ordered by sampling time. The OTUs on the Y-axis are hierarchically clustered with the Euclidean distance matrices.

**Figure 2:**
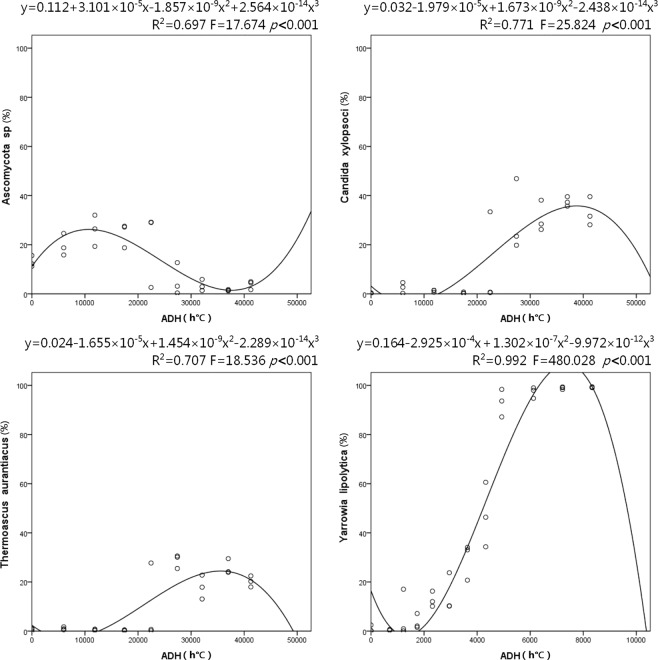
Non-linear regression curves for *Ascomycota sp*., *C. xylopsoci*, and *Thermoascus aurantiacus* on indoor cadavers and *Y. lipolytica* on outdoor cadavers. Open circles represent the relative abundances of dominant species in each sample. The cubic regression equations and the ANOVA results are shown at the top of each plot.

**Figure 3:**
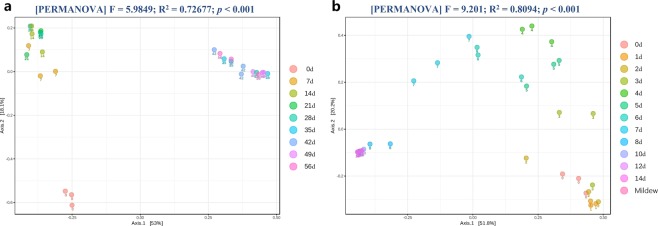
Two-dimensional PCoA plots of Bray–Curtis distance matrices for indoor (**a**) and outdoor (**b**) cadaver samples during decomposition. The statistical significance of the clustering pattern in each plot was evaluated with PERMANOVA (top). Samples obtained at various time points after death are presented in different colors.

By performing the decomposition experiment under indoor settings, we were able to explore the succession of cadaveric fungi without the confounding effects of edaphon and insects. The indoor cadavers decomposed slowly and remained intact until the dry stage, as would be the case for forensic cases occurring indoors. The sampling process lasted 56 days. During the 8-weeks decomposition period, average room temperature and humidity were 30.73 ± 5.58 °C (s.d.) and 78.39 ± 13.30% (s.d.). Accumulated degree hours (ADH) increased linearly during decomposition. The temporal succession of the cadaveric community was shown on a heat map by hierarchically clustering operational taxonomic units (OTUs) with Euclidean distance matrix (Fig. 1a). The structure of fungal communities on each indoor cadaver varied over time, with a significant shift at approximately 28 days after death. The structure of Pig c’s fungal community changed a little earlier than did fungal communities on other cadavers. The annotation results (Supplementary Fig. 1) showed a large variety of species on the torso surfaces of the antemortem pigs. During decomposition, *Candida xylopsoci*, *Ascomycota sp*., and *Thermoascus aurantiacus* gradually came to dominate, accounting for 16.38%, 13.04%, and 11.64% of the fungal community, respectively. There were significant non-linear relationships (*p* < *0.001*) between ADH and the relative abundances of these dominant species in the cubic model (Fig. 2). Box-plots of the Chao1 and Shannon indices were used to depict variation in community alpha diversity during decomposition (Supplementary Fig. 2). The Chao1 index decreased during the bloat stage (0–7 d), then increased during the decay stage (7–35 d), ultimately plateauing during the dry stage (35–56 d). The temporal trend of the Shannon index was opposite to that observed for the Chao1 index, with an increase during the bloat stage, a decrease during the decay stage, and a plateau during the dry stage. The results of principal coordinate analysis (PCoA) are presented in a two-dimensional plot for the visualization of community beta diversity. After measurement of the Bray–Curtis distance, samples obtained antemortem, 7–28 d, and 35–56 d after death formed 3 unique clusters on the plot (Fig. 3a).

### Fungal succession in outdoor cadavers

Outdoor cadaver biomass was mostly consumed by sarcosaphagous insects. As a result, the outdoor cadavers decomposed much faster than the indoor cadavers and progressed to the dry stage in only 14 days. Only parchment-like skins and skeletonized remains remained. During decomposition, mean ambient temperature was 24.85 ± 3.29 °C (s.d.); mean air humidity was 90.35 ± 8.57% (s.d.). ADH increased linearly during decomposition. The heat map created revealed a similar succession pattern in all 3 cadavers (Fig. 1b). However, the specific time points corresponding to shifts in the mycoflora population were difficult to identify in outdoor cadavers, compared with indoor cadavers. A large variety of fungal species were found on the antemortem pigs, but species diversity decreased throughout decomposition (Supplementary Fig. 3). *Yarrowia lipolytica* gradually came to dominate, accounting for 41.95% of the community present during decomposition. There was a significant non-linear relationship (*p* < *0.001*) between ADH and the relative abundance of *Y. lipolytica* in the cubic model (Fig. 2). The Chao1 index increased during the bloat stage (0–6 d), then decreased during the decay stage (6–10 d), ultimately plateauing during the dry stage (10–14 d). The Shannon index peaked during the bloat stage (0–3d), then continuously decreased (Supplementary Fig. 4). The PCoA plot showed that the community structure changed significantly during decomposition. The samples obtained at 0–2 d, 3–7 d, and 8–14 d formed 3 unique clusters, with right-to-left movement on the plot. The cluster of 3–7 d samples was more scattered than the other two clusters (Fig. 3b).

**Figure 4:**
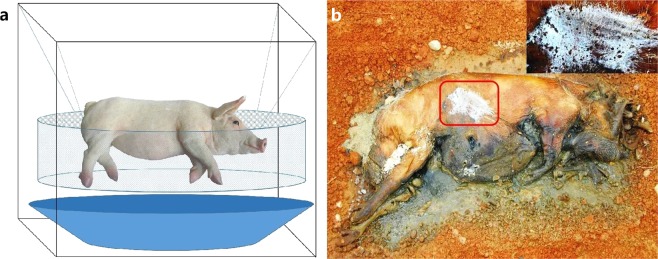
Schematic diagram of the protocols used for analysis of indoor cadavers. (**a**) Photo of an outdoor cadaver on the 10^th^ day of decomposition. (**b**) The soft tissue has been destroyed, but parchment-like skin can be seen 10 days after death in outdoor cadavers. The cadaver surface is marked with white mildew spots.

Distinctive mildew spots had formed on the torso surfaces of each outdoor cadaver by Day 10 of decomposition, which followed several days of significant rain and humidity (Fig. 4b). HTS analysis revealed that the mildew spots contained only a few species (Supplementary Fig. 5). Compared with the cadaveric communities, the Chao1 index was significantly lower for the mildew communities, while the Shannon index was significantly higher (Supplementary Fig. 6). This discrepancy likely caused by *Y. lipolytica*, which accounted for 98.88% of the mildew community (Supplementary Fig. 5). The PCoA plot also showed that Day 10 mildew samples were similar to the cadaver non-mildew samples obtained on Days 10–14 (Fig. 3b).

### Fungal succession in the soil

Cadaver decomposition resulted in significant changes to the soil mycoflora. However, the succession patterns of soil communities were difficult to discern on heat map (Fig. 1c). The annotation results (Supplementary Fig. 7) revealed a large variety of fungal species in the environmental soil 20 m away from each cadaver. However, only a limited number of species were found in the soil underneath each cadaver. *Y. lipolytica* and *Candida catenulata* gradually came to dominate, ultimately accounting for 98.09 ± 0.70% (s.d.) of the cadaveric soil communities by Day 14 of decomposition. There was a significant non-linear relationship (p < 0.001) between ADH and the total relative abundance of two dominant species in the cubic model (Supplementary Fig. 8). Other species accounted for 40.40 ± 2.75% (s.d.) at the beginning of the decomposition process, and this figure decreased over time. *Y. lipolytica* and *C. catenulata* were also found in environmental soil, but their abundance did not increase over time.

**Figure 5:**
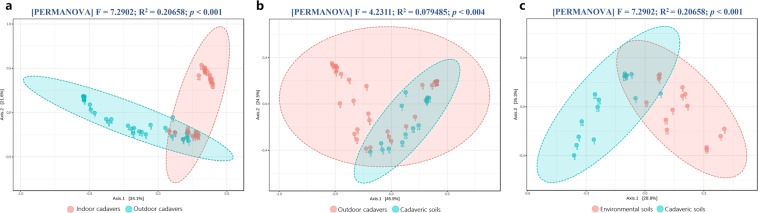
Two-dimensional PCoA plots of Bray–Curtis distance matrices for comparing samples among experimental groups. (**a**) For indoor vs. outdoor cadavers. (**b**) For outdoor cadavers vs. cadaveric soil. (**c**) For environmental vs. cadaveric soil. The statistical significance of the cluster pattern for each group was evaluated with PERMANOVA (top). The time point for each sample is presented below the dot.

Cadaveric soil communities had similar Chao1 indices to environmental soil communities, but significantly lower Shannon indices (Supplementary Fig. 9). Alpha diversity analysis suggested that cadaver decomposition did not significantly affects soil species richness but did decrease species evenness. This phenomenon was also confirmed by the variation in alpha diversity observed during decomposition (Supplementary Fig. 10). The Chao1 indices of cadaveric soil communities plateaued during the late stage of decomposition (5–14 d), but the Shannon indices decreased. The PCoA plot shows an obvious separation between cadaveric and environmental soil, except for 2 cadaveric soil samples obtained from the first day of decomposition (Fig. 5c). In the context of a forensic investigation, this significant dissimilarity in soil communities may help experts to identify the original location from which a cadaver has been moved. The results of LDA effect size (LEfSe) analysis identified 63 species with significantly differential abundance (Supplementary Table 2). Sixty species (including *Penicillium levitum*, *Saccharomycetales sp*., *Spizellomyces pseudodichotomus*, and *Ascomycota sp*.) were abundant in the communities of environmental soil. Only *Chaetothyriales sp*., *C. catenulata*, and an incertae sedis species were abundant in cadaveric soil.

### Comparisons between indoor and outdoor cadaveric communities

We further compared the fungal community composition of indoor and outdoor cadavers to determine whether the fungi that had accumulated on the torso surface could be used to identify the environment in which a cadaver has been placed. The PCoA plot in Fig. 5a shows that the fungal samples of indoor and outdoor cadavers clustered together during the early stage of decomposition but gradually separated as decomposition progressed. Samples taken from indoor cadavers on Days 0–28 were similar to those obtained from outdoor cadavers on Days 0–3. However, the 4–14 d samples from outdoor cadavers gradually shifted to the upper left of the plot, the 35–56 d samples from indoor cadavers clustered on the upper right. These data imply that the succession pattern of cadaveric fungi varies depending on the environment in which decomposition takes place. In total, 83 differentially abundant species were identified by LEfSe analysis (Supplementary Table 3). Sixty species (including *C. xylopsoci*, *T. aurantiacus*, *Ascomycota sp*., and *Trichosporon veenhuisii*) were abundant in the communities of indoor cadavers. The remaining 20 species (including *Y. lipolytica*, *C. catenulata*, *Wallemia sebi*, and *Bullera sp*.) were abundant in outdoor cadavers. On the PCoA plot of Fig. 5b, the outdoor cadaver samples obtained from the early stage of decomposition (0–3 d) were significantly different from the soil samples. However, because the cadavers decomposed in the field, the late stage samples (4–14 d) tended to be closer to the soil samples. In the annotation results, *Y. lipolytica* accounted for 35.45 ± 20.32% (s.d.) of the environmental soil community (Supplementary Fig. 7) but only 0.89 ± 0.80% (s.d.) of the community on live pigs (Supplementary Figs 1, 3). With the progression of decomposition, *Y. lipolytica* increased on outdoor cadavers but decreased on indoor cadavers. These findings suggest that the species found to be dominant during decomposition also varied among environments.

## Discussion

ITS has been selected as a target for fungal DNA barcoding and is currently used as the standard marker for analyzing the composition of fungal communities^25^. One of the spacers of the ITS region (ITS1 or ITS2) is commonly used in amplicon pyrosequencing studies. However, the use of primers targeting these regions may be accompanied by bias. Some primers (e.g. ITS1-F, ITS1, ITS5) are biased towards amplification of Basidiomycetes, whereas others (e.g. ITS2, ITS3, ITS4) are biased towards Ascomycetes^25^. Based on the comparison of ITS1 and ITS2 sequences of known taxonomic affiliations, Blaalid *et al*. reported that ITS1 and ITS2 frequently yield similar results when used as DNA metabarcodes for fungi^26^. In addition, ITS1 has been reported to represent a better DNA barcode than ITS2 for eukaryotic species because it has higher efficiency, higher diversity, shorter length of amplification products, and lower GC content^27,28^. The primers of ITS5/ITS2, which target the ITS1 region, were therefore used for amplification in the present HTS analysis of cadaveric fungi.

As decomposition progresses, fungi follow repeatable succession patterns^29^. The usefulness of fungi as a tool for PMI estimation was first reported in 1955 by Dósa, who proposed that a limited number of fungal species only present at specific PMIs^30^. Van de Voorde and Van Dijck used hyphomycetes grown on cadavers to determine that one victim had died at least 18 d prior. They proposed that fungi may be helpful in estimating PMI if the victim had died 10–20 d prior, provided that the average surrounding temperature is known^31^. We analyzed the fungal composition of postmortem communities at multiple time points after death with HTS. As would be expected the cadaveric communities examined showed significant changes during the decomposition process (Supplementary Figs 1, 3), as fungi responded to the emission of large quantities of nutrients and energy from the decomposing cadavers. Fungal communities on cadaver models underwent similar succession patterns (Fig. 1a,b). However, the patterns of succession differed significantly between indoor and outdoor environments, suggesting that the succession pattern is specific to the decomposition conditions.

During the early stage of decomposition, cadaver fungi had greater species diversity than previously reported (Supplementary Figs 1, 3)^16,17^. As decomposition progressed, several species (*Ascomycota sp*., *T. aurantiacus*, *C. xylopsoci*, and *Y. lipolytica*) came to dominate the cadaveric community. The relative abundances of dominant species showed significant non-linear relationships with ADH (Fig. 2), suggesting that these species could be used as indicators for PMI estimation. *Y. lipolytica* has previously been found on human cadavers^32^. Previous reports on cadaveric fungi included *Candida tropicalis*, *Candida glabrata*, *Candida albicansb*, and other *Candida* species but not *C. xylopsoci*^16,17^. Ascomycota has been recognized as a postputrefaction phylum in many studies^32^. For our data, the OTU annotated as *Ascomycota sp*. could not be classified to the species level using the present version of the UNITE database. *T. aurantiacus* has not been observed in other cadaver-associated studies. The discrepancy between the dominant species identified in our study and those reported previously may be attributed to differences between the microbial communities that inhabit the cadavers of humans *vs*. animals or to differences in the decomposition conditions. Additional studies are needed to determine the source of these microbial species, which may have come from the air, soil, insects, and other objects surrounding the decomposing cadavers.

As previously observed, grave soil differs physically, chemically, and biologically from its surroundings. Through the utilization of urea, ammonium (NH_4_^+^), and other nitrogenous compounds, fungi can form fruiting structures on grave soil, resulting in the release of ammonia (NH_3_) during decomposition^33^. To date, soil fungi have been found in association with decomposed human, cat, dog, crow, rabbit, snake, and kangaroo cadavers^23^. Timely surveys of the fungal species found in soil could be used to locate clandestine graves, thereby reducing the amount of time required to examine a large area^29^. Our sequencing data showed that the fungal communities in cadaveric soil were significantly different from those of environmental soil, but similar to the cadaver communities found at Days 4–14 of decomposition (Fig. 5b,c). These results suggest that decomposition can change the community structure of soil and reduce the differences between soil and cadaver communities. The effects of cadaver decomposition on soil fungi allow us to ascertain the original locations from which a cadaver has been moved.

Postmortem fungi in the grave soil have the potential to estimate PBI^23^. Previous experimental evidence suggested that the use of soil fungi for the estimation of time since burial should be based on early and late fruiting species^29^. The fruiting structures of soil macrofungi associated with the buried mammalian cadavers have been recorded repeatedly in the studies of forensic taphonomy^23^. With HTS analysis, we found that the soil communities underneath cadavers varied over time throughout the decomposition process, but the soil communities 20 m away from the cadavers did not (Supplementary Fig. 7). The introduction of decomposing mammalian cadavers into outdoor fields has a profound impact on soil communities. The communities in cadaveric soil exhibited identifiable succession patterns as decomposition progressed. *Y. lipolytica* and *C. catenulata* increased over time and accounted for the highest relative abundances in soil communities (Supplementary Fig. 7). *Y. lipolytica* is an aerobiotic and dimorphic unconventional yeast that belongs to Ascomycota, Saccharomycetes. The name “*lipolytica*” originates from its ability to hydrolyze lipids. Because this species uses hydrophobic substrates as the sole carbon source, it generally concentrates in protein- or lipid-rich environments^34^. *C. catenulata* is another yeast that belongs to Ascomycota, Saccharomycetales. This species is often found in the oral cavities and feces of living animals^31^. It is also an important early colonizer and presents strong synergistic activity with *Y. lipolytica* in protein- and lipid-rich cheese^35^. The two dominant species in soil have a synergistic responses during decomposition. A significant non-linear relationship has been found between ADH and the total relative abundance of these two species (Supplementary Fig. 8). Similar to previous studies that have used soil fungi as indicators to estimate PBI^9,23^, we propose that soil fungi underneath cadavers may be used as quantitative indicators for estimating the interval for which a cadaver has remained in the field.

The fungal communities on indoor and outdoor cadavers exhibited distinct succession patterns (Supplementary Figs 1, 3). There were similarities in the early stage of decomposition, but gradually differentiated as decomposition progressed (Fig. 5a). Janaway *et al*.^36^ reported that soil fungi may appear on the body surface “after the major phase of decomposition”, continuing that, “moulds begin to appear on the surface of the body” within the first week. The presence of soil near outdoor cadavers may contribute to the formation of atypical fungal communities^1^. We observed white mildew spots on the outdoor cadavers on Day 10 of decomposition, after several days of rainy and humid weather (Fig. 4b). These mildew spots mainly consisted of *Y. lipolytica* (Supplementary Fig. 5), which was abundant in the soil (Supplementary Fig. 7). In contrast, the indoor cadavers (not in contact with soil) did not display any mildew spots. *Y. lipolytica* was also found less frequently on indoor cadavers (Supplementary Fig. 1). We cannot yet conclude as to whether this difference between indoor and outdoor cadavers was due to the soil or other environmental factors. Nonetheless, the nature of fungi accumulation on the cadaver surface suggests that cadaver mycoflora may be used to determine the environment in which a cadaver has been placed (Fig. 5a). In-depth investigations of postmortem fungal communities that include numerous field and laboratory experiments should be performed to confirm this hypothesis.

In conclusion, our report is the first to compare the impacts of decomposition on the fungal communities under indoor and outdoor conditions. The fungal communities in these two experimental settings showed significantly different compositions and succession patterns. The succession patterns of the communities, as well as variation in the relative abundances of dominant fungi, have potential applications in PMI estimation. Differences in cadaver community structure may allow researchers to characterize the environment in which a cadaver has been placed. Additionally, the differences in soil mycoflora may be helpful for ascertaining the original location from which a cadaver has been moved. The soil fungi underneath cadavers may be used as quantitative indicators for estimating the interval for which a cadaver has remained in the field. Although there are biases and limitations in the exploration of postputrefaction mycoflora with the use of either traditional culture methods or advanced molecular approaches, our results provide an important step towards developing fungal evidence in several fields of forensic science and add to the growing body of work on the postmortem microbial community.

## Materials and Methods

### Experimental set-up

This study was approved by the Medical Ethics Committee of Xiangya Hospital, Central South University (approval number: 201503465), and followed all applicable institutional and national guidelines for the care and use of animals. In July of 2018, a total of 6 juvenile pigs (body weight: range, 7.20–8.05 kg), co-housed by the date of birth, were used for the experiments^37^. For outdoor experiments, 3 pigs were washed with distilled water and then euthanized by cranial blunt force on the following day. With a minimum between-cadaver interval of 50 m, cadavers were placed in an infertile field beside a lake (28.47°N, 112.76°E), in direct contact with soil. Plastic cages were used to protect the cadavers from scavengers. Each cage was covered with canvas to prevent disturbances in fungal colonization caused by rainfall and to prevent high body temperature and severe dehydration following exposure to direct sunlight. For indoor experiments, 3 pigs were pretreated in the same way as performed for the outdoor experiments. Cadavers were individually placed in plastic baskets, which were hung in separate cages (Fig. 4a). Putrefactive liquid that effused from the cadavers was able to drop into the basins below. To protect the cadavers from insects and contact with soil, the cages were sealed with 500 mesh gauze and placed in 3 empty cabins. All cadavers were allowed to decompose until they had dried completely. Temperature and relative humidity were recorded hourly by data loggers (MEACON Automation Technology Co., Ltd., Hangzhou, China). ADH was calculated by adding the hourly temperatures minus the minimum developmental threshold of 0 °C, starting at the time of cadaver placement^38,39^. We assessed the cadavers physical changes on a daily basis and selected the critical time points with the body-scoring system proposed by Megyesi *et al*.^40^.

### Sample collection

Fungal samples were collected from the pigs and soil. The right side of the torso surface of each cadaver, which was not in direct contact with soil, was partitioned into 4 × 4 cm^2^ areas, which were numbered consecutively. At each sampling time point, randomly selected areas on each of the 3 pigs were vigorously rubbed with sterile swabs. No sampling area was reused at any point during the study. We sampled outdoor pigs at the antemortem time point (<5 min prior to death) and at 11 postmortem time points over the course of 2 wk. We also sampled the macroscopic mildew spots that had formed on the cadaver surfaces by Day 10 after death. The indoor cadavers decomposed more slowly than the outdoor cadavers, so they were sampled prior to death, and then, after death, once a week for 8 weeks. Soil samples (depth, 0–10 cm) were collected from the area directly underneath each cadaver (cadaveric soil) and from an area 20 m away from each cadaver (environmental soil) at every stage of decomposition, as described previously^10^. All samples were individually placed in microcentrifuge tubes and stored at −80 °C until further processing. The details of each sample are shown in Supplementary Table 1.

### DNA extraction, amplification, and sequencing

Genomic DNA was extracted using a PowerSoil DNA isolation kit (MoBio Laboratories, Carlsbad, CA, USA). The cotton tip of each swab was put in a bead tube. The succeeding steps were performed as dictated by the manufacturer’s specifications. Blank swabs that had not been used for sampling were used as negative controls. DNA concentration and purity were determined via ultraviolet spectrophotometric detection and 0.8% agarose gel electrophoresis. Polymerase chain reaction (PCR) was conducted with the primers for ITS5/ITS2 (forward primer: 5′-GGAAGTAAAAGTCGTAACAAGG-3′; reverse primer: 5′-GCTGCGTTCTTCATCGATGC-3′), which targets the ITS1 region^25^. The forward primer contained a 7-bp barcode unique to each sample. All PCR reactions were conducted in 25-µL reaction volumes containing 5 µL of 5 × reaction buffer, 5 µL 5 × GC buffer, 5 µL dNTP (100 mM), 1 µL forward and reverse primer (10 µM), 2 µL DNA template, and 6 µL ddH_2_O. The thermal cycling conditions were as follows: initial denaturation at 98 °C for 2 min, followed by 30 cycles of 98 °C for 15 s, 55 °C for 30 s, and 72 °C for 30 s, with a final extension step at 72 °C for 5 min. Except for the negative controls, for which amplification failed, all amplicons were in the size range of 200–280 bp and excised from the 2% agarose gel. After quantification with the Quant-iT PicoGreen dsDNA assay kit (Thermo Fisher Scientific, Carlsbad, CA, USA), equal concentrations of the purified amplicons were pooled in a single tube. Sequencing libraries were generated using a TruSeq Nano DNA LT Library Prep Kit (Illumina Inc., San Diego, CA, USA), according to the manufacturer’s recommendations. Library quality was assessed on an Agilent Bioanalyzer 2100 system with an Agilent High Sensitivity DNA kit (Agilent Technologies, Santa Clara, CA, USA). Sequencing was conducted on the Illumina MiSeq platform, which generated 300-bp paired-end reads.

### DNA data analysis

Paired-end reads from the original DNA fragments were merged using FLASH (version 1.2.7)^41^. The sequences were processed and analyzed with QIIME (version 1.8.0)^42^. Sequences that contained ambiguous bases (N) or low-quality bases were filtered out using the QIIME filter^43^, and chimeras were removed using USEARCH (version 5.2.236)^44^. The remaining sequences were assigned to each sample in accordance with the unique barcodes. Reads were clustered into OTUs with the threshold of 97% identity with UCLUST in QIIME. A representative sequence was picked out from each OTU by selecting the longest sequence that had the highest hit number, compared to other sequences in the OTU. Representative sequences were aligned and annotated using the UNITE database (Release 5.0, https://unite.ut.ee/)^45^.

### Statistical analysis

Statistical analysis of the OTU matrix was conducted using a web-based tool, MicrobiomeAnalyst^46^. After the data were rarefied to the minimum library size, the taxonomic composition of the samples at the species level was visualized in a stacked bar plot. The relative abundance of species was calculated as the proportion of corresponding OTU sequences for the total number of sequences in a sample. A 10% relative abundance threshold was used to screen out the species that were dominant during decomposition. Non-linear regression analysis for the relative abundance of dominant species and ADH was performed with PASW Statistical Software (version 18). LEfSe analysis was used to identify species with significantly differential abundance across experimental groups. The linear discriminant analysis (LDA) included in LEfSe was used to calculate the effect size of each differentially abundant species. For the hierarchical cluster analysis, each OTU began as a separate cluster; the algorithm proceeded to combine them until all OTUs belonged to a single cluster. Distances between OTUs were measured with Euclidean and clustering algorithms using Ward’s linkage (clustering to minimize the sum of squares of any two clusters). The results were visualized as a heat map to show the temporal changes in taxonomic clusters. The alpha diversity of the community in each sample was estimated with Chao1 and Shannon indices. The Chao1 index assesses the taxon richness of the community by inferring the number of rare organisms that may have been lost due to undersampling^47^. The Shannon index, which assesses taxon richness and evenness, was used to describe the actual diversity of a community^48,49^. The statistical significance of differences in alpha diversity between experimental groups was estimated using t-test/ analysis of variance (ANOVA). Beta diversity analysis provides a way to determine the degree of diversity dissimilarity between communities. Dissimilarity was first measured as Bray–Curtis distance^50^. The ordination-based method of PCoA was used to visualize the Bray–Curtis distance matrix in a two-dimensional plot. The statistical significance of the clustering pattern in the ordination plot was evaluated by permutational multivariate analysis of variance (PERMANOVA).

## Data Availability

Total raw sequencing data were published in the Sequence Read Archive (SRA) under Accession Number PRJNA516254.

## Supplementary information

Supplementary Info
