# Supplementary material for: Fungal succession during mammalian cadaver decomposition and potential forensic implications
Source: Sci Rep. 2019 Sep 9;9:12907. doi: 10.1038/s41598-019-49361-0 (PMC6733900; doi:10.1038/s41598-019-49361-0)
Supplement: Supplementary file 1 — Supplementary Info [file 41598_2019_49361_MOESM1_ESM.pdf]

Fungal succession during mammalian cadaver decomposition and potential forensic implications

Xiaoliang Fu, Juanjuan Guo, Dmitrijs Finkelbergs, Jing He, Lagabaiyila Zha, Yadong Guo & Jifeng Cai*

Department of Forensic Science, School of Basic Medical Sciences, Central South University, Changsha, Hunan 410013, China.

***Corresponding author**

Jifeng Cai

Department of Forensic Medicine, School of Basic Medicine Sciences, Central South University, Tongzipo Road 172, Changsha, Hunan 410013, PR China.

Tel.: +86-731-82650414,

Fax: +86-731-82650414

E-mail address: cjf_jifeng@163.com

Supplementary Figures and Tables

Supplementary Figures

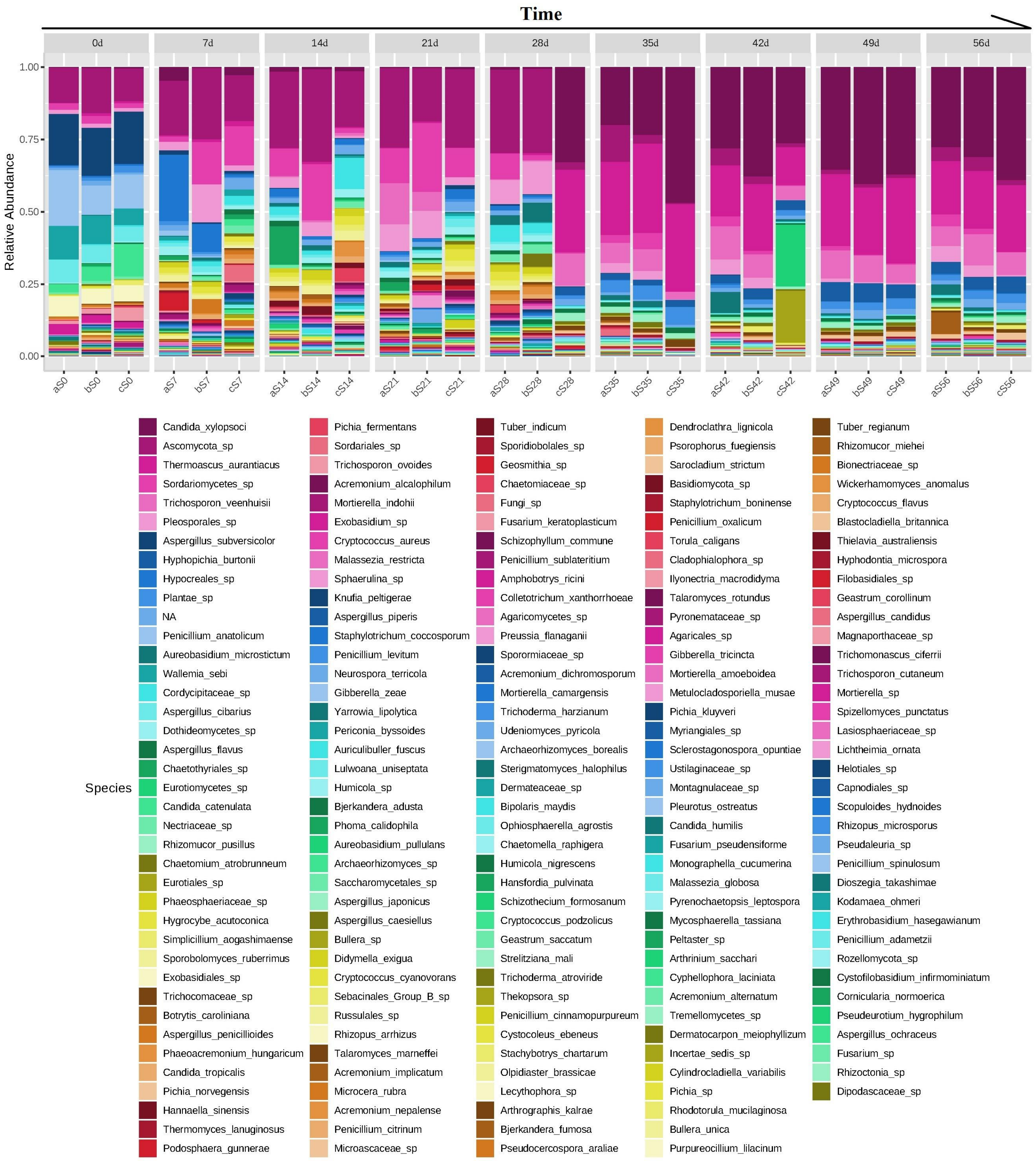

Supplementary Figure 1 The taxonomic composition of the fungal communities on indoor cadavers during decomposition. The samples are arranged sequentially on the X-axis according to sampling time. Each of the stacked bars shows the species-level fungal composition of each sample.

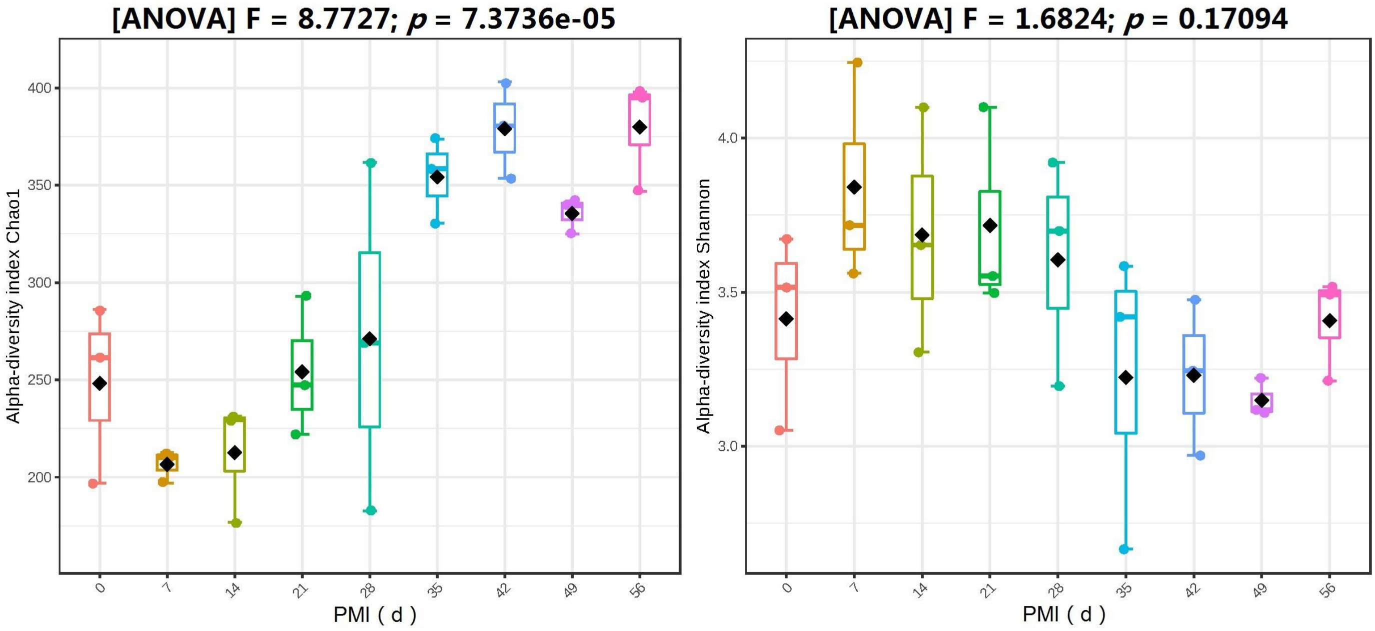

Supplementary Figure 2 The alpha diversity variation of fungal communities on indoor cadavers. Samples from each time point were measured with Chao1 and Shannon indices, as shown in each boxplot. The ANOVA results are shown at the top of each plot.

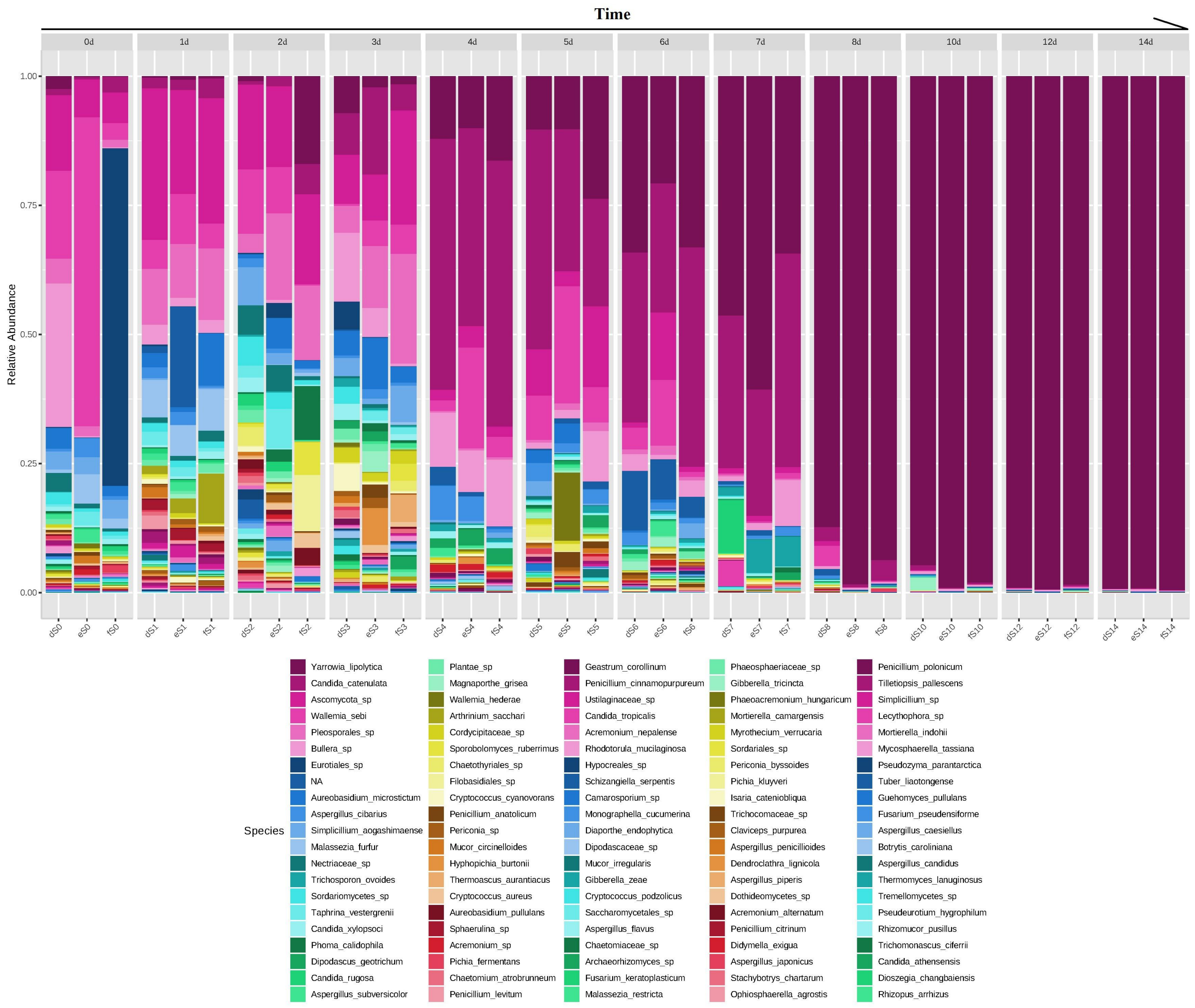

Supplementary Figure 3 The taxonomic composition of the fungal communities on outdoor cadavers during decomposition. The samples are arranged sequentially on the X-axis according to sampling time. Each of the stacked bars shows the species-level fungal composition of each sample.

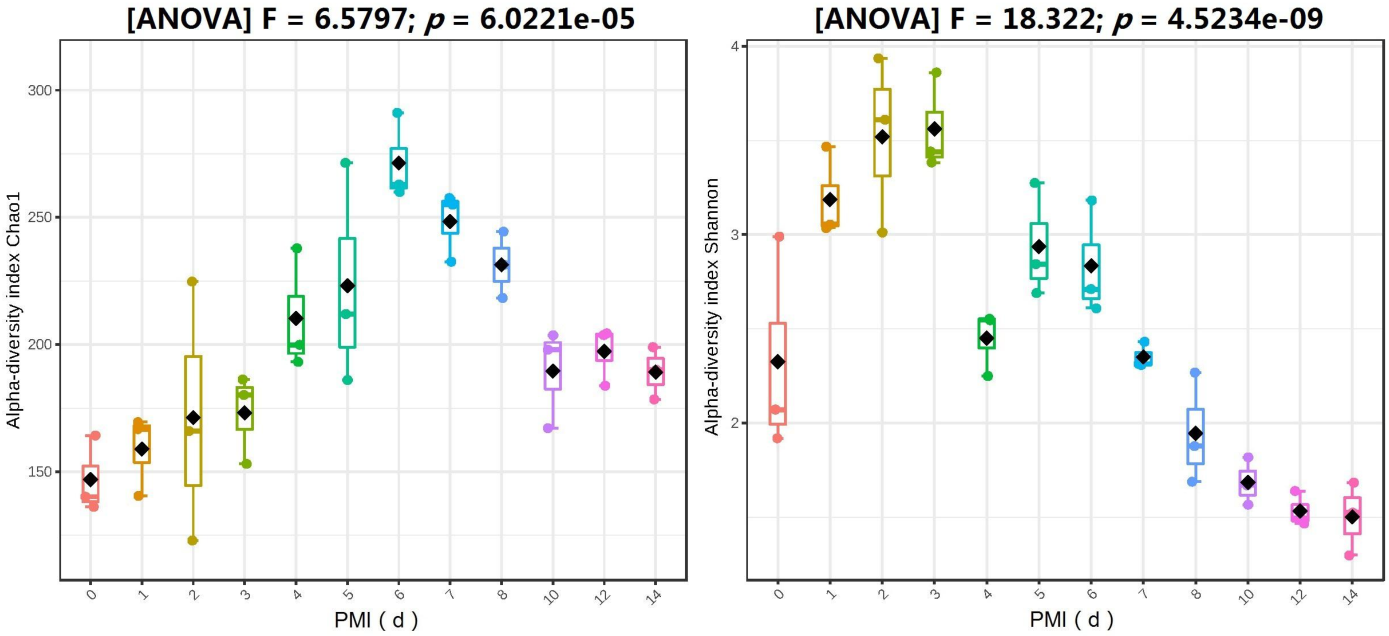

Supplementary Figure 4 The alpha diversity variation of the fungal communities on outdoor cadavers. Samples from each time point were measured with Chao1 and Shannon indices, as shown in each boxplot. The ANOVA results are shown at the top of each plot.

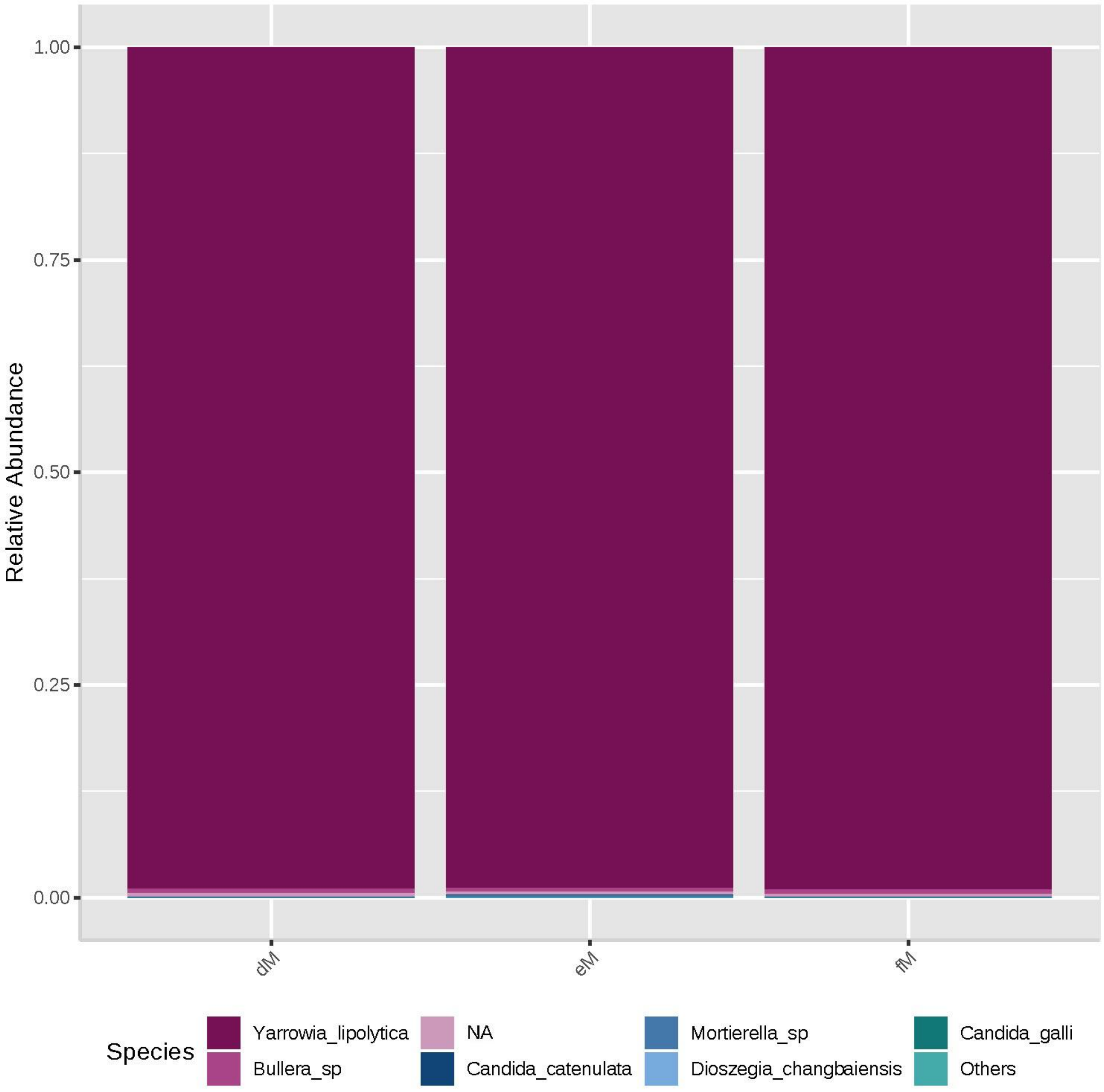

Supplementary Figure 5 The taxonomic composition of the fungal communities in mildew spots found on outdoor cadavers on the 10th day of decomposition. Each of the stacked bars shows the species-level fungal composition of each mildew sample.

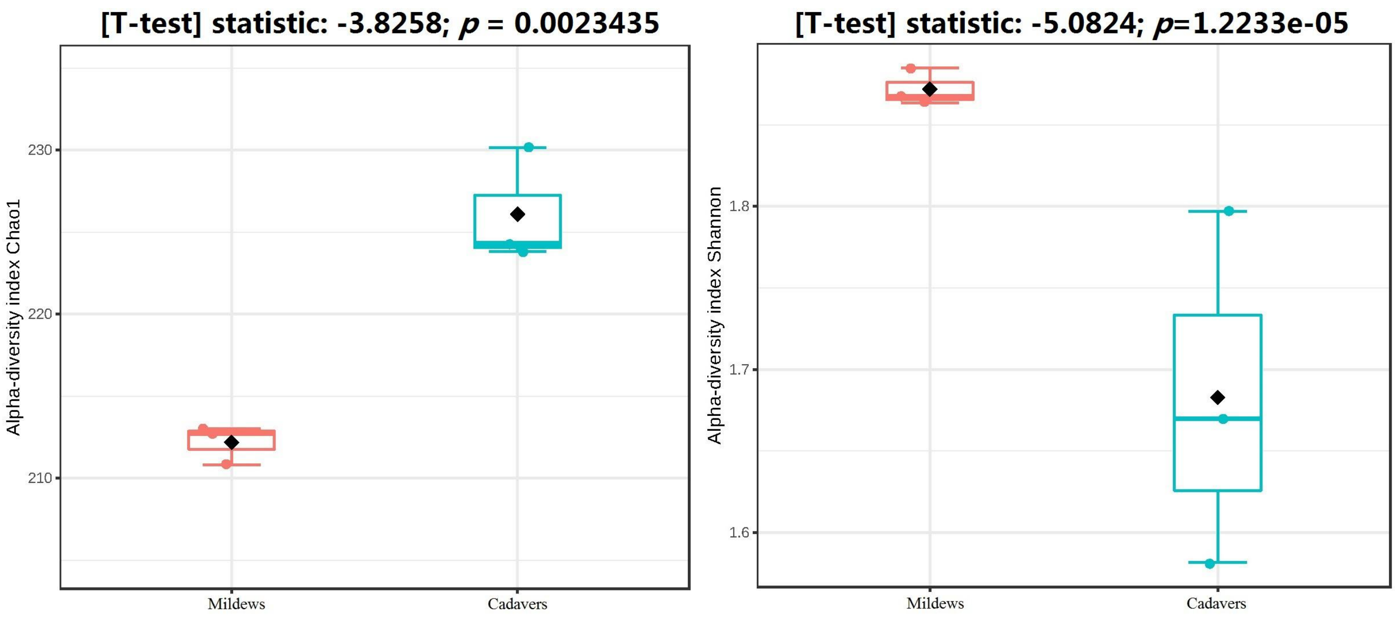

Supplementary Figure 6 Alpha diversity of fungal communities on cadaver surfaces and mildew spots. Each sample was measured with Chao1 and Shannon indices, as shown in each boxplot. The t-test results are shown at the top of each plot.

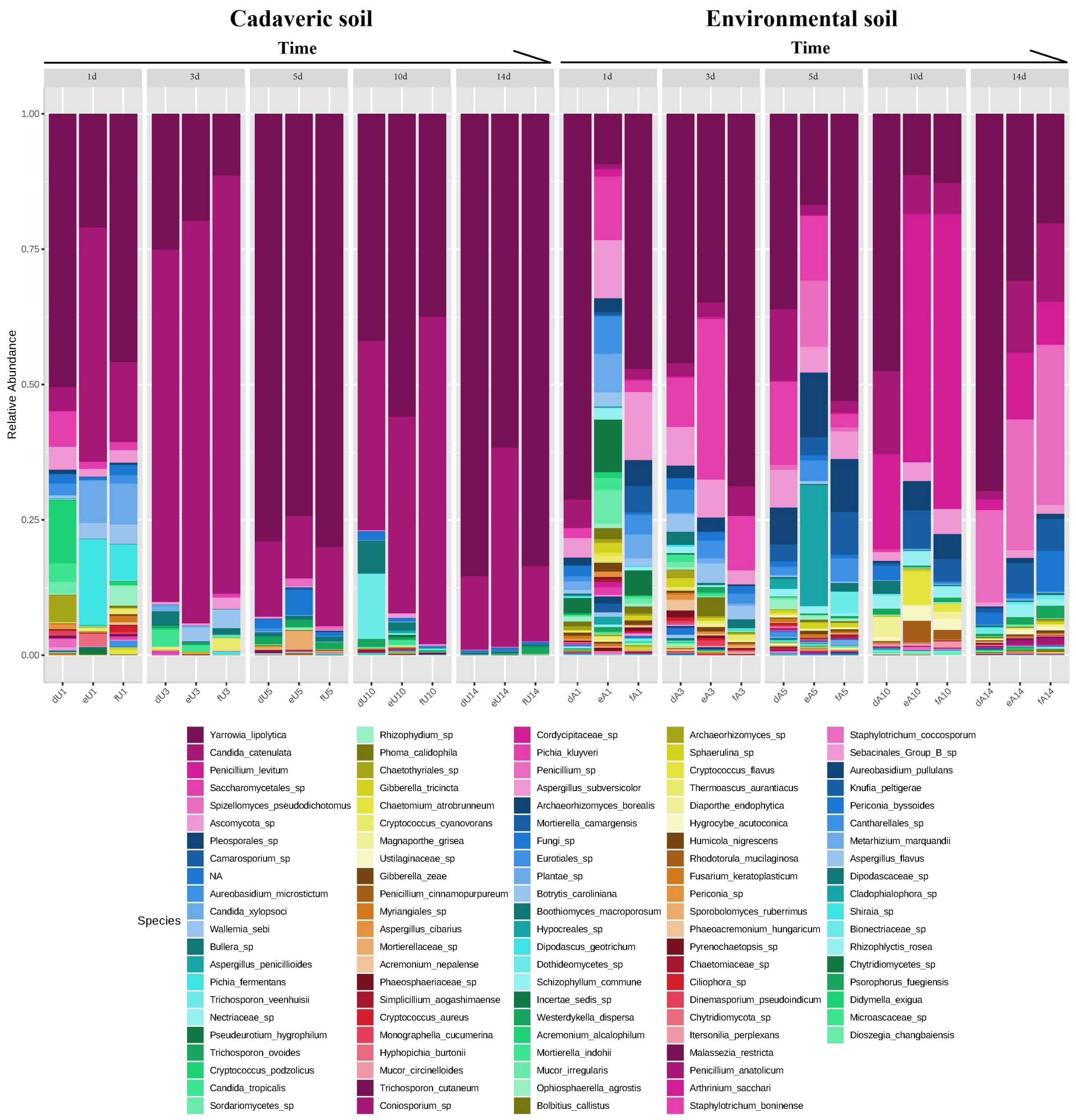

Supplementary Figure 7 The taxonomic composition of fungal communities in cadaveric and environmental soil during decomposition of outdoor cadavers. The samples are arranged sequentially on the X-axis according to sampling time. Each of the stacked bars shows the species-level fungal composition of each soil sample.

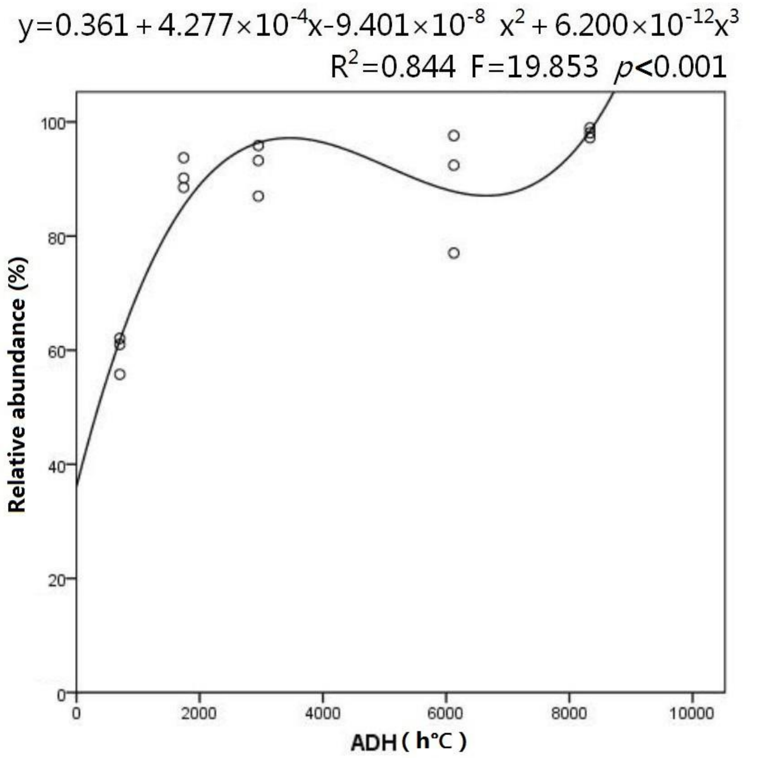

Supplementary Figure 8 Non-linear regression curves for the dominant species in cadaveric soil communities. Each open circle represents the total relative abundance of *Y. lipolytica* and *C. catenulata* in each sample. The cubic regression equations and the ANOVA results are shown at the top of each plot.

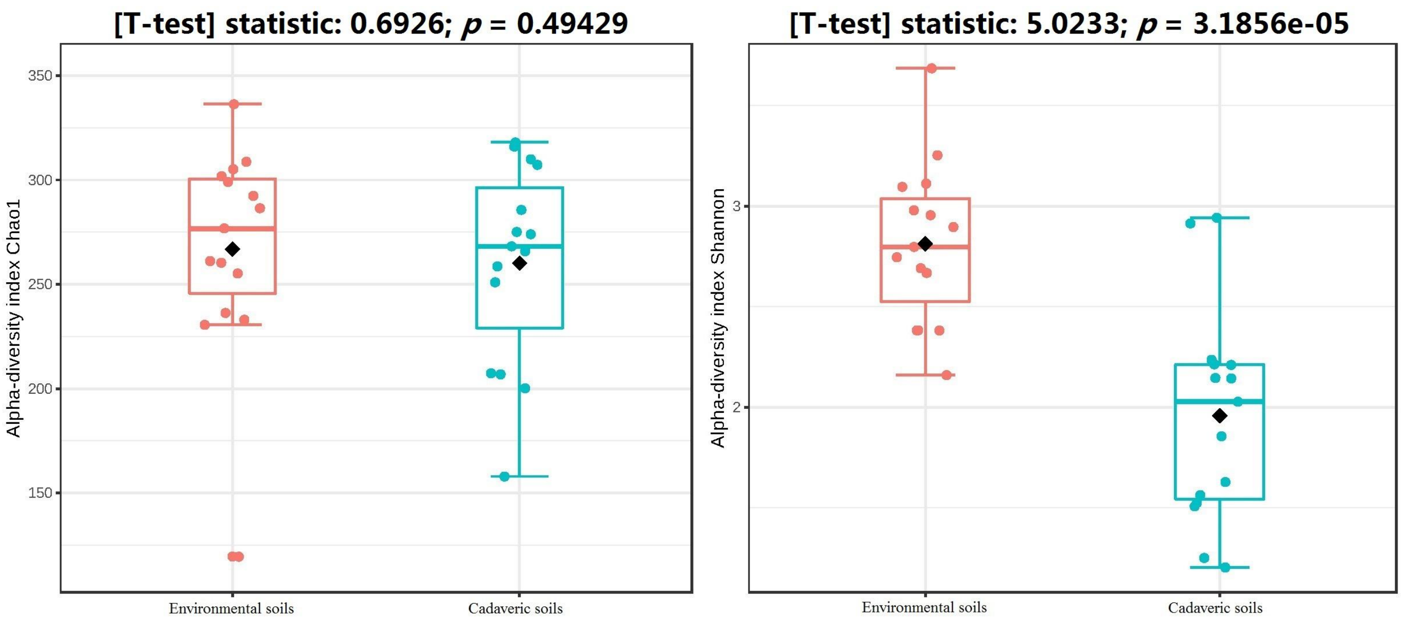

Supplementary Figure 9 Comparison of alpha diversity in soil communities underneath the cadavers (cadaveric soil) vs. near the cadavers (environmental soil). Each sample was measured with Chao1 and Shannon indices, as shown in each boxplot. The t-test results are shown at the top of each plot.

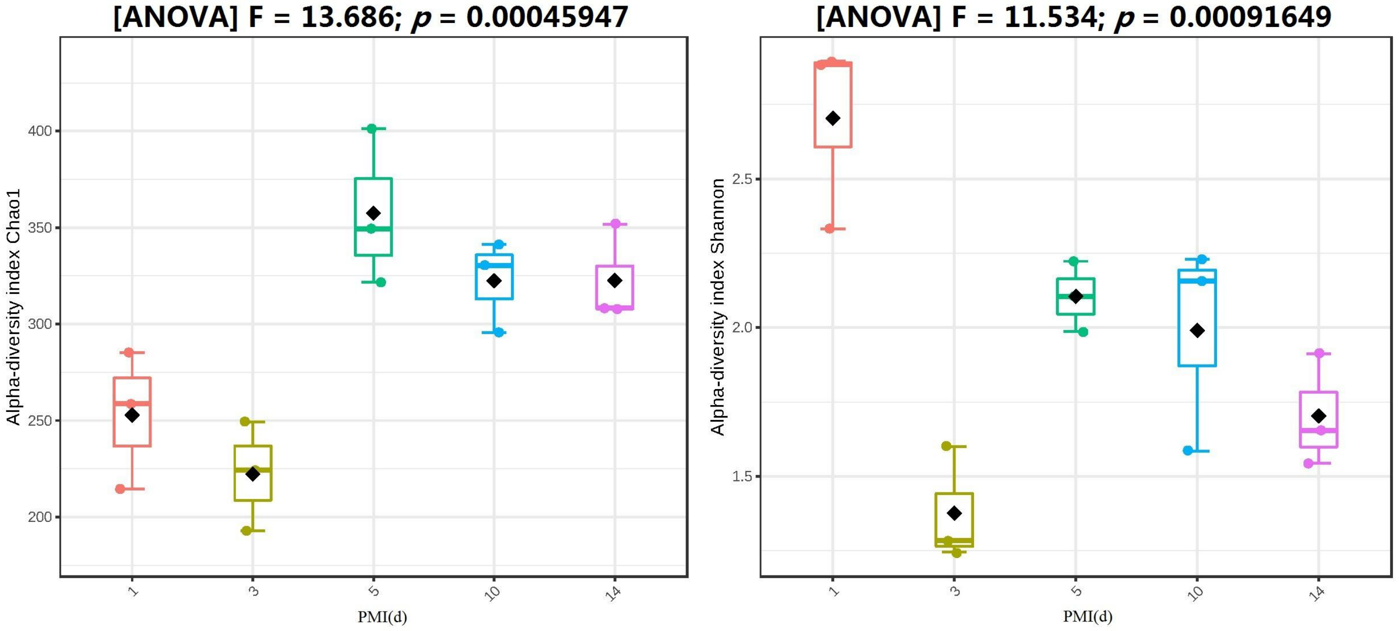

Supplementary Figure 10 The alpha diversity variation of cadaveric soil communities. Samples from each time point were measured with Chao1 and Shannon indices, as shown in each boxplot. The ANOVA results are shown at the top of each plot.

Supplementary Tables

Supplementary Table 1 Fungal samples obtained from the cadavers and soil.

Sample names	Environments	Sample subjects	Sampling time (d)	Average daily temperature (℃)	Average daily humidity (%)	ADH (℃)	Raw sequences	Clean sequences	Observed Species	Chao1	Shannon
aS0	Indoor	Cadavers	Prior to death	-	-	-	47767	46553	144	164.00	2.83
bS0							43860	42350	99	146.77	3.34
cS0							59798	57941	114	197.56	3.26
aS7			7	36.20	65.30	5996.2	38867	37538	126	102.00	3.30
bS7							47233	46295	94	132.86	3.27
cS7							36676	35241	105	116.17	3.83
aS14			14	34.30	72.00	11863.8	61262	59480	131	124.50	3.27
bS14							50226	48757	91	131.00	2.92
cS14							50710	47719	125	93.25	3.66
aS21			21	31.30	79.30	17441.1	44471	43118	144	135.33	3.17
bS21							53225	51178	125	131.88	3.01
cS21							42181	39239	109	116.00	3.68
aS28			28	26.30	89.70	22457.6	66810	63610	130	100.00	3.34
bS28							41625	39020	124	133.00	3.38
cS28							62930	62253	106	105.75	2.29
aS35			35	28.90	77.90	27405.3	54568	54179	106	116.38	2.79
bS35							45436	44888	89	107.36	2.64
cS35							54023	53742	129	92.46	1.86
aS42			42	28.80	87.70	32071.9	65819	64518	175	141.00	2.75
bS42							48759	48364	107	111.50	2.43
cS42							54208	53821	93	115.83	2.24
aS49			49	23.60	80.90	37007.6	47566	46961	111	101.50	2.38
bS49							51891	51277	102	89.27	2.14
cS49							53868	53068	82	92.67	2.15
aS56			56	26.30	81.80	41256.1	62704	61717	100	128.67	2.77
bS56							62860	61771	91	138.10	2.71
cS56							61371	60626	106	107.11	2.27
dS0	Outdoor	Cadavers	Prior to death	-	-	-	57938	55363	118	125.33	3.18
eS0							73154	72163	134	109.00	1.83
fS0							77998	76521	109	128.25	1.94
dS1			1	28.21	85.24	702.7	47477	46231	116	142.67	3.32
eS1							47762	46719	164	141.60	3.02
fS1							54305	52846	152	139.17	2.95
dS2			2	21.98	94.49	1224.5	52386	48462	201	112.75	3.90

eS2							51930	50105	164	144.86	3.58
fS2							67421	65891	161	200.44	2.98
dS3							49656	46859	147	127.77	3.73
eS3			3	21.26	87.66	1739.5	48719	47866	127	156.36	3.41
fS3							51966	49648	117	128.14	3.34
dS4							67297	66775	103	190.40	2.48
eS4			4	23.83	83.85	2315.5	63818	63256	126	128.71	2.45
fS4							67299	66835	137	157.00	2.22
dS5							48030	47449	144	169.11	2.78
eS5			5	26.51	84.62	2951.4	51585	50776	119	166.65	2.56
fS5							47412	46595	146	222.00	3.21
dS6							55857	55601	186	244.13	2.70
eS6			6	28.31	87.18	3633.5	27881	27318	172	198.00	3.08
fS6							52460	51982	120	209.58	2.59
dS7							73741	73466	111	196.80	2.29
eS7			7	28.96	82.10	4322.9	62111	61679	100	219.22	2.30
fS7							50401	50244	108	182.50	2.33
dS8							52448	52265	114	202.05	2.22
eS8			8	25.17	93.25	4928.5	70587	70555	130	139.84	1.66
fS8							39667	39631	178	170.38	1.83
dS10							59078	58997	111	169.78	1.77
eS10			10	24.31	97.69	6124.2	67920	67898	137	123.50	1.61
fS10							50147	50102	183	161.00	1.54
dS12							89072	89014	188	156.64	1.45
eS12			12	22.50	95.78	7216.8	63350	63339	168	111.05	1.62
fS12							65718	65697	146	142.12	1.44
dS14							78986	78964	140	134.65	1.48
eS14			14	24.80	88.45	8334.5	73857	73840	127	130.14	1.67
fS14							103299	103268	115	137.00	1.28
dU1							36056	35592	151	168.65	2.77
eU1			1	28.21	85.24	702.7	49070	48993	167	197.94	2.11
fU1							37321	36945	188	215.72	2.79
dU3							19504	19211	128	163.05	1.46
eU3			3	21.26	87.66	1739.5	55462	55361	97	133.91	1.14
fU3							23457	23363	93	104.18	1.15
dU5		Cadaveric soil					69932	69789	194	218.57	2.08
eU5			5	26.51	84.62	2951.4	76863	76703	186	226.32	1.79
fU5							71672	71592	169	206.13	1.89
dU10							60753	60695	159	204.04	2.15
eU10			10	24.31	97.69	6124.2	96700	96357	178	204.45	2.04
fU10							91341	91130	157	220.14	1.43
dU14			14	24.80	88.45	8334.5	77497	77445	140	162.66	1.60

eU14							85713	85642	160	201.76	1.80
fU14							62802	62774	134	175.05	1.50
dA1		Environmental soil	1	28.21	85.24	702.7	64998	64653	177	215.08	2.52
eA1							41089	40178	107	135.50	3.62
fA1							59820	59038	159	207.24	3.10
dA3			3	21.26	87.66	1739.5	44544	43242	155	190.00	3.14
eA3							49790	49181	160	199.05	2.67
fA3							38343	37951	163	193.52	2.27
dA5			5	26.51	84.62	2951.4	57563	56622	217	258.58	2.95
eA5							67960	66935	164	193.53	2.75
fA5							60088	59466	199	234.25	2.64
dA10			10	24.31	97.69	6124.2	74240	73965	204	233.20	2.58
eA10							74376	74167	141	180.60	2.20
fA10							61506	61335	151	194.24	2.02
dA14			14	24.80	88.45	8334.5	68977	68670	196	230.73	2.15
eA14							39493	39139	195	217.16	2.70
fA14							45117	44839	187	209.88	2.91
dM		Mildew spots	10	24.31	97.69	6124.2	73446	73431	99	123.80	1.78
eM							83369	83354	95	104.63	1.80
fM							64903	64894	92	102.22	1.79

Supplementary Table 2 LEfSe analysis was performed to identify soil species with significantly differential abundance with regard to the presence of cadavers, followed by linear discriminant analysis (LDA) to calculate the effect size (LDA score) for each differentially abundant species. Species were considered to be significant if the adjusted p-value ≤ 0.05.

	p-values	FDR	Environmental soil	Cadaveric soil	LDA score
Penicillium levitum	1.43E-05	0.00030218	950510.6668	3470.168382	5.68
Saccharomycetales sp.	0.014584	0.032206	632871.9587	69127.33152	5.45
Spizellomyces pseudodichotomus	0.020166	0.041108	565124.8078	14551.04697	5.44
Ascomycota sp.	8.85E-05	0.00085289	489530.3443	85295.16148	5.31
Pleosporales sp.	6.56E-06	0.00026721	367404.0774	10173.90276	5.25
Camarosporium sp.	4.86E-05	0.00062878	293544.6981	6348.83079	5.16
Aureobasidium microstictum	0.00026848	0.0018973	208722.7414	30955.47932	4.95
Aspergillus penicillioides	0.0012618	0.0058299	164832.9981	3667.33704	4.91
Nectriaceae sp.	1.71E-05	0.00030218	120982.6886	3627.903308	4.77
Pseudeurotium hygrophilum	0.030454	0.056153	116921.0142	3549.035845	4.75
Gibberella tricincta	1.01E-05	0.00026721	62108.12729	2642.060018	4.47
Phoma calidophila	0.00010943	0.00096666	62975.66939	5441.854963	4.46
Chaetomium atrobrunneum	0.014455	0.032206	56193.06755	1183.011948	4.44
Ustilaginaceae sp.	0.00020948	0.0015861	51894.7908	0	4.41
Magnaporthe grisea	0.001512	0.0061642	52249.69439	1537.915533	4.4
Penicillium cinnamopurpureum	0.0023512	0.0089011	43101.06865	512.638511	4.33
Sordariomycetes sp.	0.032476	0.057375	62502.46461	20071.76939	4.33
Gibberella zeae	2.87E-05	0.00043418	45151.6227	3549.035845	4.32
Phaeosphaeriaceae sp	0.0046526	0.014091	23660.23897	2918.096139	4.02
Coniosporium sp.	0.0019923	0.0078217	19164.79356	276.0361213	3.98
Acremonium nepalense	0.03106	0.056153	21254.78134	4258.843014	3.93
Mucor circinelloides	0.007701	0.019436	17390.27564	1971.686581	3.89
Archaeorhizomyces borealis	0.0072844	0.019436	15418.58906	591.5059742	3.87
Fungi sp.	0.00088173	0.005055	14196.14338	315.4698529	3.84
Mortierella camargensis	0.017389	0.037616	14275.01084	591.5059742	3.84
Cordycipitaceae sp.	0.0072419	0.019436	16483.29981	3075.831066	3.83
Plantae sp.	0.0071096	0.019436	13052.56516	709.8071691	3.79
Dothideomycetes sp.	5.39E-06	0.00026721	11278.04724	394.3373161	3.74
Myriangiales sp.	0.0098642	0.023764	20899.87776	12066.72187	3.65
Rhizophydium sp.	0.034157	0.059355	43022.20119	34070.74411	3.65
Mucor irregularis	0.024367	0.046963	7650.143933	118.3011948	3.58
Schizophyllum commune	0.0093973	0.023166	8833.155882	1419.614338	3.57
Ophiosphaerella agrostis	5.43E-05	0.00062878	7452.975275	157.7349265	3.56
Hygrocybe acutoconica	7.85E-06	0.00026721	6703.734374	78.86746323	3.52
Diaporthe endophytica	0.0035716	0.011831	6822.035569	276.0361213	3.52
Mortierella indohii	0.0041022	0.013177	7058.637959	473.2047794	3.52
Acremonium alcalophilum	0.0011149	0.0058299	7255.806617	946.4095587	3.5
Thermoascus aurantiacus	0.0012177	0.0058299	6703.734374	433.7710478	3.5

Archaeorhizomyces sp.	0.029181	0.055235	6664.300643	670.3734374	3.48
Ciliophora sp.	0.000553	0.0034481	5205.252573	0	3.42
Sporobolomyces ruberrimus	0.00051964	0.0034426	4732.047794	236.6023897	3.35
Humicola nigrescens	0.0026871	0.0098218	5165.818841	1143.578217	3.3
Cryptococcus aureus	0.019881	0.041108	11948.42068	8044.481249	3.29
Arthrinium sacchari	0.00090608	0.005055	3351.867187	78.86746323	3.21
Penicillium anatolicum	0.0050299	0.01481	3154.698529	157.7349265	3.18
Chytridiomycota sp.	0.031255	0.056153	2957.529871	118.3011948	3.15
Sebacinales Group B sp.	0.0032604	0.011149	2918.096139	157.7349265	3.14
Knufia peltigerae	5.93E-05	0.00062878	2247.722702	39.43373161	3.04
Staphylotrichum coccosporum	0.036836	0.061978	2799.794945	630.9397058	3.04
Geastrum saccatum	0.001375	0.0058299	2089.987776	0	3.02
Aureobasidium pullulans	0.0075482	0.019436	1892.819117	0	2.98
Periconia byssoides	0.011317	0.026658	1932.252849	39.43373161	2.98
Aspergillus flavus	0.0075482	0.019436	1813.951654	0	2.96
Chytridiomycetes sp.	0.0030882	0.010912	1616.782996	157.7349265	2.86
Metarhizium marquandii	0.022794	0.045588	1498.481801	118.3011948	2.84
Cladophialophora sp.	0.0044682	0.01393	1340.746875	39.43373161	2.81
Shiraia sp.	0.001375	0.0058299	1222.44568	0	2.79
Microascaceae sp.	0.0013582	0.0058299	1104.144485	0	2.74
Bionectriaceae sp.	0.012192	0.028094	1064.710754	78.86746323	2.69
Staphylotrichum boninense	0.036598	0.061978	1892.819117	1025.277022	2.64
Chaetothyriales sp.	0.018243	0.038676	29259.82886	36436.76801	-3.56
Incertae sedis sp.	0.023442	0.046017	670.3734374	9266.926929	-3.63
Candida catenulata	0.00012469	0.0010167	624748.61	3432903.506	-6.15

Supplementary Table 3 LEfSe analysis was performed to identify cadaveric species with significant differential abundance with regard to the environment in which decomposition took place, followed by linear discriminant analysis (LDA) to calculate the effect size (LDA score) for each differentially abundant species. Species were considered to be significant if the adjusted *p*-value ≤ 0.05.

	p-values	FDR	Indoor cadavers	Outdoor cadavers	LDA score
Candida xylopsoci	8.50E-06	4.71E-05	1670852.079	55757.64804	5.91
Thermoascus aurantiacus	3.57E-09	1.20E-07	1230334.634	28657.37704	5.78
Ascomycota sp.	0.0026982	0.0070644	1379993.507	699852.9032	5.53
Trichosporon veenhuisii	1.50E-08	2.70E-07	508831.3312	2700.088126	5.4
Sordariomycetes sp.	2.91E-07	3.50E-06	558194.9055	61422.86362	5.4
Hypocreales sp.	8.85E-06	4.72E-05	191358.3928	19579.78015	4.93
Hyphopichia burtonii	0.00057349	0.0017953	202887.6034	30810.82156	4.93
Plantae sp.	5.24E-06	3.26E-05	185461.2678	43880.57328	4.85
Cordycipitaceae sp.	0.0051767	0.011397	140007.0235	40004.37315	4.7
Aspergillus flavus	4.10E-09	1.20E-07	95281.63741	10104.62427	4.63
Dothideomycetes sp.	1.44E-06	1.30E-05	95612.93657	11628.60039	4.62
Hygrocybe acutoconica	5.95E-06	3.43E-05	82559.7498	579.7735239	4.61
Rhizomucor pusillus	1.16E-07	1.67E-06	79401.36451	1275.501753	4.59
Chaetomium atrobrunneum	1.50E-05	7.21E-05	84238.3322	18768.09722	4.52
Phaeosphaeriaceae sp.	0.00053836	0.0017228	83641.99371	17012.21169	4.52
Trichocomaceae sp.	2.03E-06	1.72E-05	69418.21659	7686.140431	4.49
Botrytis caroliniana	1.06E-05	5.44E-05	64470.81586	2186.574433	4.49
Aspergillus penicillioides	0.00011053	0.00038821	59965.14733	5880.560028	4.43
Phaeoacremonium hungaricum	0.024429	0.044529	60075.58038	8000.87463	4.42
Hannaella sinensis	1.40E-07	1.84E-06	46889.87395	579.7735239	4.36
Candida tropicalis	2.25E-06	1.80E-05	65221.76061	19281.61091	4.36
Pleosporales sp.	0.019594	0.037125	382341.3132	340211.1038	4.32
Thermomyces lanuginosus	4.17E-09	1.20E-07	40131.37116	2020.924855	4.28
Mortierella indohii	0.0018034	0.0050919	40285.97743	3445.511228	4.27
Acremonium alcalophilum	1.14E-05	5.65E-05	36244.12772	1739.320572	4.24
Knufia peltigerae	1.94E-05	8.73E-05	26437.67269	198.7794939	4.12
Malassezia restricta	0.00010779	0.00038806	30280.74291	8878.817395	4.03
Aspergillus piperis	5.44E-06	3.26E-05	26680.6254	6493.463468	4
Neurospora terricola	0.0039661	0.0091758	21644.87823	1987.794939	3.99
Nectriaceae sp.	0.0095103	0.02014	88302.26852	69109.00405	3.98
Bjerkandera adusta	3.46E-09	1.20E-07	17845.98123	281.604283	3.94
Periconia byssoides	0.0033433	0.0083539	23544.32672	7470.795979	3.91
Staphylotrichum coccosporum	1.44E-08	2.70E-07	20496.37448	4671.318107	3.9
Auriculibuller fuscus	3.63E-05	0.00015361	17890.15445	1838.710319	3.9
Sebacinales Group B sp.	4.23E-06	2.90E-05	16277.83189	1424.586373	3.87
Rhizopus arrhizus	1.42E-06	1.30E-05	14466.72983	579.7735239	3.84
Russulales sp.	0.016434	0.032715	14731.76916	1838.710319	3.81
Microcera rubra	0.01221	0.025118	14599.2495	2203.139391	3.79

Aspergillus japonicus	0.020102	0.037593	17382.16241	5300.786504	3.78
Cryptococcus aureus	0.021717	0.040093	33306.60853	22329.56315	3.74
Gibberella zeae	8.56E-05	0.00031592	22506.25603	11727.99014	3.73
Acremonium implicatum	0.0010144	0.0030432	12103.46252	1888.405192	3.71
Sporidiobolales sp.	1.70E-05	7.91E-05	10734.09267	695.7282287	3.7
Aspergillus caesiellus	0.0024241	0.0065861	12302.24201	2782.912915	3.68
Sphaerulina sp.	0.0033845	0.0083539	32224.36462	22594.60247	3.68
Talaromyces marneffei	0.00014068	0.00048235	11838.42319	2600.698379	3.66
Tuber indicum	0.0012084	0.0035512	10756.17928	1623.365867	3.66
Agaricomycetes sp.	1.42E-08	2.70E-07	8061.612808	248.4743674	3.59
Humicola nigrescens	0.0034808	0.0083539	7509.447548	1209.241921	3.5
Strelitziana mali	0.00096191	0.0029471	6891.022455	679.1632709	3.49
Lulwoana uniseptata	2.69E-05	0.00011719	5742.518713	298.1692409	3.44
Archaeorhizomyces sp.	0.017711	0.034006	16763.73732	11479.51577	3.42
Talaromyces rotundus	2.79E-06	2.11E-05	4660.274802	165.6495783	3.35
Staphylotrichum boninense	0.0032985	0.0083539	5433.306167	1225.806879	3.32
Trichoderma atroviride	0.011615	0.02424	6846.849235	2666.95821	3.32
Metulocladosporiella musae	5.20E-06	3.26E-05	3335.078176	16.56495783	3.22
Humicola sp.	0.0001892	0.0006336	3290.904955	430.6889035	3.16
Myriangiales sp.	0.0018392	0.0050932	4174.369372	1954.665023	3.05
Pyrenochaetopsis leptospora	0.016954	0.032991	3268.818344	1060.157301	3.04
Lichtheimia ornata	0.0012544	0.0036128	1855.275276	414.1239456	2.86
Kodamaea ohmeri	4.01E-05	0.00016488	1258.936795	347.8641143	2.66
Fusarium keratoplasticum	0.016585	0.032715	8591.691459	7702.705389	2.65
Mortierella camargensis	0.034874	0.062773	8017.439588	7586.750684	2.34
Saccharomycetales sp	0.039715	0.068904	17558.8553	17658.24504	-1.7
Dioszegia changbaiensis	0.00030702	0.0010048	0	646.0333552	-2.51
Acremonium sp.	0.0040144	0.0091758	19171.17786	20358.33317	-2.77
Penicillium polonicum	0.016529	0.032715	728.8581443	5333.91642	-3.36
Myrothecium verrucaria	0.0034527	0.0083539	154.606273	7371.406232	-3.56
Mucor irregularis	0.0037985	0.008967	132.5196626	8381.86866	-3.62
Dipodascaceae sp.	0.0025691	0.0068509	44.17322087	11098.52174	-3.74
Camarosporium sp.	0.0051298	0.011397	132.5196626	12142.11409	-3.78
Rhodotorula mucilaginosa	0.0052236	0.011397	2562.04681	18718.40234	-3.91
Eurotiomycetes sp.	0.03969	0.068904	94309.82655	119648.6904	-4.1
Mucor circinelloides	6.43E-05	0.00025018	198.7794939	25824.76925	-4.11
Periconia sp.	3.82E-06	2.75E-05	110.4330522	29966.00871	-4.17
Magnaporthe grisea	3.81E-07	4.22E-06	198.7794939	44079.35277	-4.34
Dipodascus geotrichum	4.84E-05	0.00019341	110.4330522	52245.87698	-4.42
Eurotiales sp.	0.0072941	0.015677	81389.15945	137224.1106	-4.45
Taphrina vestergrenii	6.84E-05	0.00025919	1082.243911	59551.02338	-4.47
Bullera sp.	7.69E-07	7.91E-06	12633.54117	241235.4808	-5.06
Wallemia sebi	0.0372	0.066134	138284.2679	628242.5905	-5.39
Candida catenulata	1.15E-07	1.67E-06	87816.36309	1349580.244	-5.8

Yarrowia lipolytica	3.85E-09	1.20E-07	25664.64132	4211673.657	-6.32
